# Experiences of females on the autism spectrum through the perspective of minority stress theory: a review

**DOI:** 10.3389/fpsyt.2025.1578963

**Published:** 2025-07-18

**Authors:** Aleksandra Grzeszak, Ewa Pisula

**Affiliations:** Department of Health and Rehabilitation Psychology, Faculty of Psychology, University of Warsaw, Warsaw, Poland

**Keywords:** autism, females, life experiences, mental health, minority stress, social experiences, stigma

## Abstract

**Introduction:**

People on the autism spectrum, especially females, are at high risk of co-occurring psychiatric and psychological conditions, suicidal ideation, and psychiatric hospitalization. The minority stress theory offers a framework for understanding mental health disparities by focusing on the influence of social factors. The current review aims to synthesize the literature on the experiences of autistic females in terms of the unique, chronic and socially based stressors that emerge at the intersection of sex/gender and autism.

**Methods:**

We conducted a literature search in a systematic way on the Web of Science and Scopus databases, applying the specified inclusion and exclusion criteria. We chose a narrative manner of data presentation.

**Results:**

Based on the evidence from 104 studies of various methodologies, we show that the burdens encountered by autistic females are more than the sum of strains common for the females and for autistic individuals. Females on the autism spectrum face also specific challenges related to their not meeting social gendered expectations and the stereotypical image of autism.

**Discussion:**

This intersectional disadvantage can enhance our understanding of the adverse mental health outcomes observed in this population. We emphasize key directions and provide methodological recommendations for future research in this area. Additionally, we underscore the importance of enhancing practitioners' understanding of the unique characteristics of female autism and the specific vulnerabilities faced by this group to improve support and outcomes.

## 1 Introduction

Autism spectrum disorder (ASD) is defined as a complex neurodevelopmental condition, characterized by lifelong challenges in social communication and interpersonal interactions, as well as repetitive, rigid patterns of behavior and interests (1). Since the identification of autism is based only on behavioral indicators, its conceptualization has evolved with the accumulation of data. Diagnostic criteria have been broadened, including different patterns of traits grouped under the term autism spectrum (2, 3). This has contributed to an increase in the frequency of diagnoses of ASD, amongst others in female individuals (3).

The male-to-female ratio in the autistic population is commonly reported as 3-4:1 (4). Increasingly, it is recognized that this asymmetry may be due to the underdiagnosis of girls and women, rather than an accurate reflection of the number of females on the autism spectrum (3–5). Nonetheless, females remain a minority within the diagnosed population. Consequently, the experiences of autistic females have been the subject of minimal research until recently, when the literature on this topic has begun to expand considerably. At this stage, there is a need to review prior research and chart directions for future scientific inquiry.

### 1.1 The minority stress and autism

The minority stress model (6) is an elaboration of the social stress theory, which links mental health disparities with disadvantaged social status. Social stressors are understood as stigma-related experiences encountered by socially marginalized groups, compounded by a deficiency of coping resources (7). In following sections, we provide the rationale behind the application of this theory in analyzing the data on autistic females, and the overview of the model.

#### 1.1.1 The applicability of the social stress theory

The premise of the social stress theory is the existence of health disparities between disadvantaged and advantaged groups (7). Being on the autism spectrum is linked with poorer mental (8) as well as physical health outcomes compared to the general population (9). These health inequities contribute to the observed premature mortality in the autistic population due to chronic illnesses, medication side effects, or suicide (10, 11). Numerous studies have demonstrated a heightened risk of co-occurring anxiety disorders, depressive disorders, suicidal tendencies, and psychiatric hospitalization among females on the autism spectrum (12–17). Additionally, evidence suggests that autistic females experience poorer physical health (18).

The neurodiversity movement originated in autistic self-advocacy groups and is based on the assumption of equality and appreciation of differences in the human brain functioning (19). It promotes the notion that autistic individuals are an integral part of society and that autism should be understood not solely as a medical condition but also as an essential component of personal identity (19, 20). The neurodiversity movement draws attention to the social situation of people on the spectrum – employing the term “neurominority”. This term refers to a part of the population that shares not only challenges related to the way their nervous system works, but also experiences of social marginalization (21). Consequently, employing theories that analyze stigma-related processes may provide a deeper understanding of autistic individuals’ life experiences and their effects on mental health (19, 21–23).

The stigmatization process begins with the labeling of a person and attribution of negative stereotypes to the label (24). Examples of unfavorable stereotypes applied to autistic individuals include “weird”, “loner”, “violent”, or “retarded” (25–28). Positive associations, such as high intelligence or exceptional skills in specific domains, also exist (27, 29). In addition to the label itself, some behaviors that are common for autism but contrary to neurotypical social norms (e.g., inadequate facial expressions or tone of speaking, stimming, poor eye contact, not following the rules of conversation, etc.) are stigmatized. Some research indicates that stigma towards these behaviors is even greater than stigma towards the label of autism itself (30, 31).

Gender-related factors have a major influence on the formation of social structures and institutions, and prejudices and stereotypes play important roles in this process (32). The theory of intersectionality posits that disadvantaged identities do not simply combine additively, but they interact dynamically and reciprocally, producing a specific constellation of experiences (7, 33). While primarily used to analyze the experiences of Black females (33), the intersectionality framework enhances our understanding of challenges faced by females on the autism spectrum (34), including Black autistic females (35).

#### 1.1.2 The minority stress model – an overview

The minority stress model was primarily used to study sexual and gender minorities (6), but has been shown applicable in disability studies (36) and autism research (21). This theoretical framework states that minorities face unique and persistent challenges that are rooted in social hierarchies and go beyond general life stressors (6).

Minority stressors range from external (distal) to internal (proximal) in relation to the individual. The most distal are the actual events of discrimination and/or victimization. These events can manifest as individual acts of discrimination or violence in interpersonal relations, such as social exclusion or bullying, which are commonly experienced by autistic persons (37, 38). Additionally, there are more permanent situations in relation to institutions that are reported by individuals on the autism spectrum. These include instances of lack of necessary accommodations in the workplace or educational setting (39–41), as well as unmet healthcare needs (42, 43).

Living in a stigmatizing environment can affect individuals even in the absence of a concrete event of discrimination or victimization (6, 44). Expectation of rejection, a more proximal stressor, involves fear, insecurity, and vigilance that negatively impact self-concept and mental health (6, 45). It has been shown that autistic people anticipate negative reactions of others (21, 25, 46).

Concealing one’s minority identity is yet another, even more proximal stressor (6). Given that certain minority identities, such as autism, are not immediately discernible, individuals may employ concealment as a means of safeguarding themselves from violence and discrimination. Concealment of autism can be understood as hiding the fact of diagnosis as well as behaving in such a way as to pass as non-autistic (22, 47–51). The latter phenomenon is known as masking or camouflaging, and is widely recognized as more prominent in females than in males on the autism spectrum, with the influence of gender stereotypes and higher social motivation considered among the possible explanations for the difference (5, 48–50, 52) Masking of autistic traits is one of the factors that may account for under-diagnosis of females (4, 5). The broad topic of camouflaging and its consequences has been elaborated in many works and reviews (5, 22, 47, 49–51) and is beyond the scope of this paper. These two strategies of stigma management are often utilized jointly; however, disclosing one’s diagnostic status does not necessarily lead to abandoning all masking behaviors (22, 53). Besides, most of those who disclose do so selectively and the question of if and to whom to disclose the diagnosis is a psychologically tasking dilemma (25, 44).

Internalized stigma is conceptualized as the most proximal stressor (6). Due to awareness of stigma and exposure to prejudice-related events, the individual incorporates into their self some negative judgments that are harmful to their self-concept and self-esteem (6, 54). While individuals on the autism spectrum exhibited lower levels of internalized stigma compared to patients with serious mental illness (55, 56), studies specifically designed to investigate this phenomenon among autistic individuals have demonstrated that negative perceptions about autism influence the way they think about themselves (25, 26, 44).

The minority stress model assumes that the influence of stressors on mental health outcomes is modified by minority identity – its prominence, valence, and integration with other identities, as well as coping and social support resources arising from the identity (6). As indicated by Botha (57) in her qualitative study aiming to create the first measure of autistic community connectedness, autistic identity enables one to be part of a community, and this community reciprocally helps to develop a positive identity. Cooper, Cooper, Russell, and Smith (58) found that positive valuation of autism is positively linked to higher collective self-esteem in individuals who identify with the autistic community.

Botha and Frost (21) were the first to use the social stress framework to study potential causes of poor mental health and well-being outcomes in the autistic population. Utilizing adapted scales of minority stress, they demonstrated that despite controlling for general stress exposure, minority stress significantly predicted poorer well-being and higher psychological distress in individuals on the autism spectrum. Their study provides preliminary results that may indicate a relationship between minority stress and gender, but the data did not allow for further exploration (21).

### 1.2 Aim of the review

In the late 2000s, research into the topic of sex/gender differences in autism presentation saw a notable increase (59, 60) and, starting with the important work of Cridland et al. in 2013 (61), the area of research on the life experiences of autistic females has grown substantially. The purpose of this review is to elucidate findings on the experiences of females on the autism spectrum, focusing on unique, chronic, and socially determined stressors resulting from the intersection of sex/gender and autism. We chose the narrative review approach, as it enables the extraction of information from a variety of studies not necessarily connected directly to minority stress theory and the synthesis thereof in the context of the theory (62).

## 2 Method

Despite choosing the narrative manner of data presentation, we approached the literature search in a systematic way. The first author conducted the search on the Web of Science and Scopus databases using the queries containing combinations of alternative terms referring to: (1) autism, (2) sex/gender differences, (3) experiences, stigma, and minority stressors, linked with Boolean operators. A detailed presentation of the keywords used is shown in **Table 1**.

**Table 1:** Search terms used in the review.

Sex/gender	Autism	Experiences, stigma, minority stressors
women OR females OR girls OR “sex difference*” OR “gender difference*”	autistic OR “on the autism spectrum” OR “with autism” OR “with ASD” OR “with Asperger’s” OR neurodivergent OR autis* OR Asperger*	experience* OR stigma* OR label* OR stereotype* OR prejudice* OR rejection* OR discrimination OR inequit* OR inequalit* OR sexis* OR victimization OR violence OR bullying OR concealment OR disclosure OR identity

**Figure 1:**
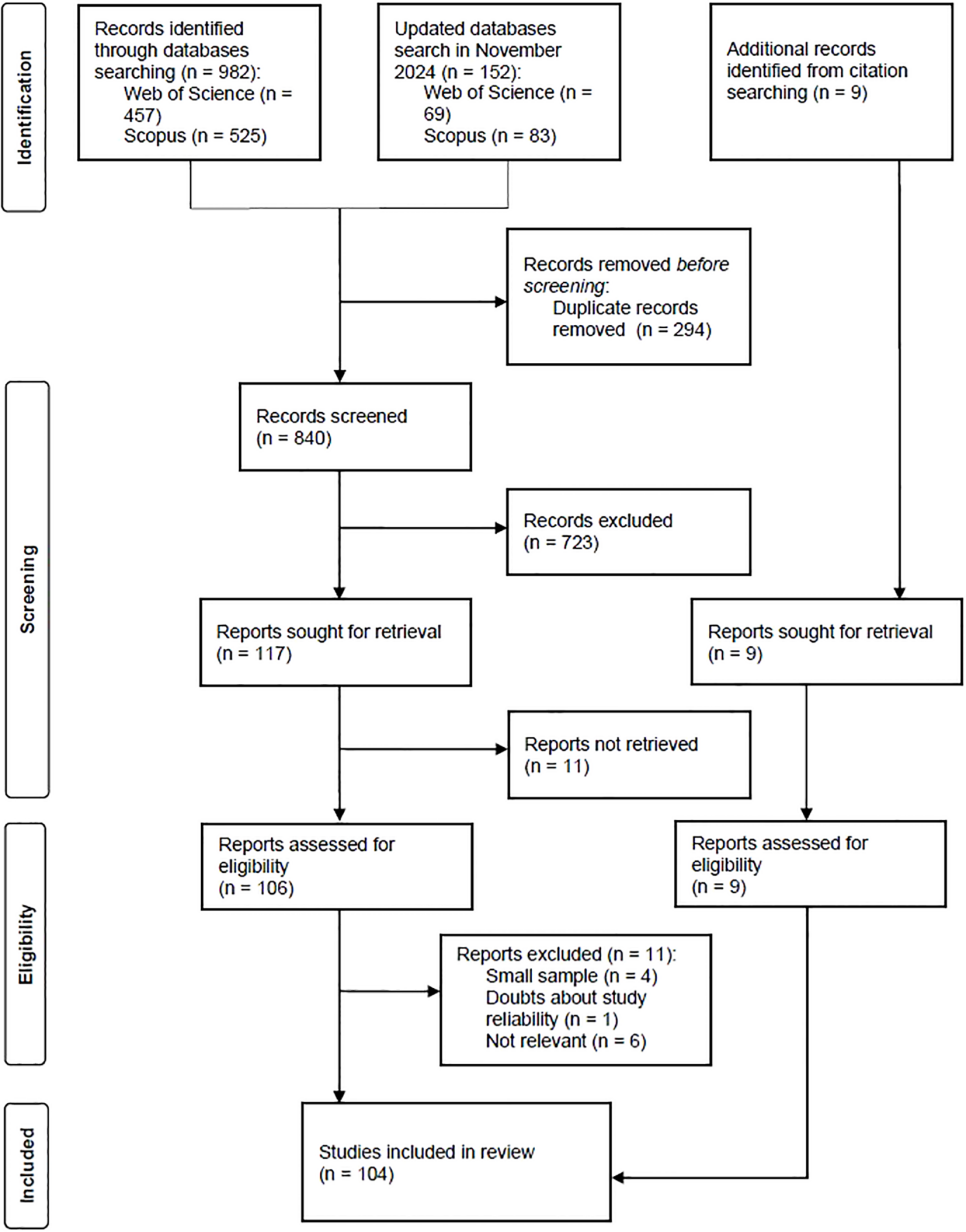
PRISMA flow diagram © 2021 by Page MJ, et al., licensed under CC BY 4.0.

The literature search was conducted on 12-13 February 2024 and was narrowed to original research articles published in English in peer-reviewed journals since 2013. Additional search was undertaken on 19 November 2024, aimed at supplementing the query with works published in the meantime. We included studies involving females with formal autism diagnoses as well as self-diagnosed females. We decided to incorporate studies researching children, adolescent, and adult populations because the minority status of autistic females appears early in their lives and affects identity, mental health and well-being since then. We included articles describing quantitative, qualitative, and mixed methods studies. The records were pre-screened for duplicates, then the first author screened titles and abstracts. The exclusion criteria at this stage were: (1) research without female autistic participants or with the number or female participants not allowing to check for sex differences; (2) studies in which autistic women were an indistinguishable part of a wider sample; (3) case studies; (4) research exclusively on the symptoms or autistic traits presentation; (5) research focusing on parents’ or caregivers’ experiences; (6) research testing psychometric tools; (7) research solely on the prevalence of mental and physical health problems. The last criterion stems from the assumptions of minority stress theory. This theory applies to explain disparities in mental health related to social factors (that is, social stressors) rather than all differences. Since the purpose of our study was to review research on those experiences of women on the autism spectrum that can be considered minority stressors, we only included studies that examined the relationship between mental health and experiences. The prevalence of mental health problems is the subject of other reviews (8, 16). We decided to exclude also the studies focused on camouflaging of autistic traits. The PRISMA flow chart illustrates the reasons for exclusion of retrieved records for which the full text has been assessed (**Figure 1**). Any doubts about the inclusion of articles were resolved collaboratively by us. In the end, a total of 104 articles were included in the review.

## 3 Autistic females’ social experiences and mental health

Two methodological approaches are commonly used to examine minority stress theory. Between-group analyses focus on examining the effect of social status on mental health by comparing the prevalence of psychiatric symptoms or disorders in minority and non-minority groups. Within-group analyses investigate the relations between stress processes (e.g., levels of exposure to stigma-related experiences or coping resources) and mental health outcomes (7). Only six quantitative studies incorporated in our review included mental health measures as one of the variables. A summary of the results of these studies is presented in **Table 2**.

**Table 2:** Quantitative studies that included mental health measures as one of the variables (*n =* 6).

Authors and year	Country	Sample	Topic – type of experience	Mental health measure	Relevant findings about mental health
Cazalis, Reyes, Leduc, & Gourion (2022) (68)	France	women of the French autistic community (*n* = 225), age > 18	Sexual victimization	Question about co-occurrence of psychiatric disorders Question about reported consequences of the assault in the next 6 months	Participants who have encountered sexual violence demonstrated higher prevalence of mental health problems, particularly PTSD
DaWalt et al. (2020) (67)	United States	high school students with educational classification of autism, (*n* = 547; 76 females, 471 males), age 14-21; parents’ reports	Social participation	Question about current mental health diagnoses	Females demonstrated higher prevalence of internalizing disorders (the variable of presence of mental health diagnoses was not included in further analyses on social participation rates)
Greenlee, Winter, & Marcovici (2020) (66)	United States	adolescents with autism diagnosis (*n* = 105; 50 females, 55 males), age 13-17, and their primary caregivers	Peer victimization	Revised Children’s Anxiety and Depression Scale (RCADS) – Short Version, subscales Anxiety and Depression	Girls reported more symptoms of anxiety and depression than boys Relational violence predicted anxiety symptoms in girls, but not in boys
Hampton, Allison, Baron-Cohen & Holt (2023) (63)	United Kingdom	384 autistic and 490 non-autistic mothers, age > 18	Childbirth and postnatal experiences	Question about being diagnosed with postnatal depression Question about being diagnosed with postnatal anxiety	Postnatal depression and anxiety were more prevalent in autistic than in non-autistic mothers (items on postnatal depression and disorders were not included in further multivariate analyses)
O’Connor, van den Bedem, Blijd-Hoogewys, Stockmann, & Rieffe, (2022) (65)	Netherlands	pre-adolescents and adolescents: autistic (*n* = 104; 18 girls, 84 boys) and non-autistic (*n* = 202; 111 girls, 91 boys), age 9-16	Friendship	Child Depression Inventory Child Symptom Inventory (CSI) – generalized anxiety disorder subscale	Autistic participants reported more depression and anxiety symptoms, no effect of gender In all groups depression negatively correlated with positive friendship quality (PFQ) and positively with negative friendship quality For the whole sample, anxiety correlated negatively with PFQ, but in sub-sample of autistic females anxiety and PFQ correlated positively
Pohl et al. (2020) (64)	Participants from different countries (mostly Western)	355 autistic and 132 non-autistic mothers	Pregnancy, childbirth, and motherhood	Question about having additional psychiatric or psychological diagnosis Question about experiencing prenatal depression Question about experiencing postnatal depression	Additional psychiatric or psychological diagnosis were more prevalent in autistic mothers More autistic than non-autistic mothers reported experiencing prenatal and postnatal depression

### 3.1 Between-group comparisons: autistic vs non-autistic females, autistic females vs autistic males

Three studies compared the occurrence of psychiatric diagnoses or internalizing symptoms between autistic and non-autistic groups. In two studies on perinatal and motherhood experiences, mothers on the autism spectrum demonstrated higher prevalence of perinatal depression (63, 64) and postnatal anxiety (63) than non-autistic mothers. Moreover, other psychiatric problems were found to be more prevalent in autistic than non-autistic mothers (64). In these two studies, mothers on the autism spectrum also reported more negative experiences with professional care. There were no within-groups analyses testing whether more negative experiences with perinatal care providers were related to more adverse mental health outcomes (63, 64). In research on friendship quality, autistic pre-adolescents and adolescents exhibited more symptoms of depression and anxiety than their non-autistic peers (65).

Three studies explored mental health differences between males and females on the autism spectrum. While O’Connor et al. (65) found no gender differences in rates of internalizing symptoms, Greenlee, Winter, and Marcovici (66) identified higher levels of depression and anxiety among autistic girls experiencing peer victimization compared to autistic boys. Another study reported that female adolescents were more likely than males to have diagnoses of internalizing disorders according to parental reports (67).

The studies mentioned above add to the body of research that indicates that females on the autism spectrum may be at greater risk of psychiatric disorders, compared to non-autistic females (63–65) and autistic males (66, 67). However, the only study incorporating both gender and neurotype variables found significant differences between neurotypes but no gender differences in rates of depression and anxiety symptoms (65). Of note, this study involved adolescents diagnosed by clinicians in accordance with formal criteria and only 18 girls.

### 3.2 Within-group analyses: social experiences and mental health

Three studies examined the relationships between social experiences and mental health. Sexual victimization heightened the number of co-occurring mental health problems – precisely post-traumatic stress disorder (PTSD) was more prevalent in autistic females who had survived sexual assault in comparison to those who had not have such experience (68). Bullying, specifically relational violence, predicted symptoms of anxiety in girls, but not in boys on the autism spectrum (66). Depression and anxiety correlated negatively with positive friendship experiences and positively with negative friendship experiences in autistic and non-autistic (pre-)adolescents. Only autistic girls had a positive correlation between anxiety and positive friendship experiences (65). The results of these studies suggest that experiencing victimization is associated with poorer mental health (66, 68). Therefore, the relationship between socially-based experiences and mental health outcomes is marked. Interestingly, even if there are no differences in rates of these experiences between females and males on the autism spectrum, there are differences in relations between those experiences and mental health symptoms. Autistic females may be more prone than autistic males to detrimental effects of relational violence (66). Additionally, in this group, even engagement in positive social interactions may be paid for with more immense anxiety (65). Although only the study of O’Connor et al. (65) included both between-group and within-group analyses, all findings presented above support the potential value of analyzing the nature and the extent of social stressors faced by females on the autism spectrum. In the next part of this work, we will outline a landscape of possible relations between autism, sex/gender, social environment, and wellbeing and mental health, based on the currently available data. The structure of the following text mirrors Meyer’s minority stress processes, starting from events external to the individual, gradually approaching identity-related experiences and ending with experiences that may modify the impact of minority stressors on mental health.

## 4 Discrimination

### 4.1 Diagnosis and support services

Females obtain autism diagnosis later than males (52, 69–71), even if they do not differ from males in levels of autistic characteristics (71). There are many females, who were not diagnosed until adulthood and they believe that this delay had prevented them from accessing adequate support and harmed their well-being (29, 72–82). Late-diagnosed autistic females describe the road to diagnosis as long and winding due to clinicians’ stereotypical, male-centric view of autism and their dismissive attitude towards women seeking diagnosis (29, 73, 75, 76, 78, 80, 81, 83–87). Moreover, being primarily diagnosed with other condition is another factor that hinders autism diagnosis in females (29, 70, 75, 77, 78, 82, 84, 85). Also, more intensive camouflaging, observed to a greater extent in females than in males, was associated with a later time to diagnosis (52, 71), and this relationship may be stronger in women than in men, although further research on larger samples is needed (71). In qualitative studies, autistic females indicate that this was one of the barriers to both referral for diagnosis (29, 85), and during the diagnostic process if providers did not create conditions for feeling safe enough to “unmask” (80, 87). Females also report facing greater barriers to accessing post-diagnosis support services than males (88) and frequently evaluate these services as inadequate or insufficient (79, 80, 89, 90).

### 4.2 Education

Lack of adequate support in educational settings is reported retrospectively by females diagnosed later in life (76, 80, 86, 91) but also by students with formal diagnosis (92, 93). They cite teachers’ lack of understanding, pressure on academic achievements and attendance, and insufficient accommodations as significant obstacles to reaching their full academic potential (80, 86, 91–93). Autistic females reported not receiving adequate support not only in learning, but – even more frequently – in emotional and social functioning (72, 86) and the percentage of females dissatisfied with the level of support in their educational experiences was significantly higher than in the male sample (72). Sometimes, an unaccommodating mainstream school environment leads to exclusion from school to alternative settings which provide a more individualized learning environment. However, such settings often host many students with emotional, behavioral, and social difficulties, making them potentially challenging for vulnerable autistic girls (93, 94).

### 4.3 Employment

Research suggests that females and males on the autism spectrum do not differ significantly in rates of employment (95–97) or in average hourly wages (95). Although the evidence of sex/gender differences in average weekly work hours in autistic adults population are ambiguous (95, 96), females tend to prefer part-time work (72, 96) or even withdrawal from the job market, a choice not necessarily linked with parenting responsibilities (96). More pronounced differences are noted between autistic and non-autistic females, with autistic females less likely than the general female population to have paid employment (72). Autistic females are also more likely to experience job instability, underemployment, difficulties in gaining and maintaining jobs, and negative work experiences compared to non-autistic females, but there were no significant differences between autistic females and males (97, 98). These negative employment experiences can therefore be considered common to all people on the spectrum (97).

Differences between males and females on the autism spectrum in vocational experiences may be subtle. For instance, co-occurring anxiety and depression, although less common in males, were found to be a greater barrier to employment in this group (95). In turn, autistic females report a stressful mismatch of their social skills and preferences with stereotypical gendered expectations formulated by coworkers and superiors (72, 99–101).

### 4.4 Healthcare

To date, no significant gender differences have been found in the rates of unmet medical services among autistic adults (88). However, healthcare needs among females on the autism spectrum are more likely to go unmet compared to non-autistic females. The findings concerning perinatal, and perimenopausal care indicate that professionals’ lack of knowledge about autism and dismissive or even stigmatizing attitudes towards autistic individuals translate into lower quality of care, poorer treatment outcomes, and higher stress and anxiety (64, 75, 102–108). These barriers are also faced by females on the autism spectrum who experience eating disorders, hindering their recovery (109, 110).

## 5 Victimization

Research on victimization examines various forms of violence and uses different methodologies. The data from register-based studies indicate that females with autism diagnosis are at increased risk of child maltreatment (111), and serious physical violence in adolescence and adulthood (112) compared to non-autistic individuals and males with autism diagnosis. Self-report studies show that being a victim of physical violence is most prevalent among autistic females compared to both non-autistic females and autistic males (113, 114). In contrast, other self-report study suggests the importance of diagnosis rather than sex/gender in the experience of various forms of victimization (115). The two most researched forms of victimization encountered by people on the spectrum are bullying and sexual violence.

### 5.1 Bullying

Autistic status, but not sex/gender, was found to be associated with higher rates of experiencing recurrent and intentional social aggression in educational and work settings (72, 97, 100, 115–119). While the overall rates of peer victimization do not appear to differ between females and males on the autistic spectrum, there are some nuances. Autistic males may be rejected and victimized more blatantly, while peer aggression between females may consist of less pronounced behaviors such as ignoring or exclusion (66, 120, 121). In their narratives, autistic females describe various form of peer violence: physical (86, 93, 94, 122), verbal (61, 77, 84, 86, 90, 93, 94, 100, 123), and relational (29, 61, 74, 81, 85, 91, 122, 124–126). They point out their divergence from social norms, especially gender norms, as causes of experienced bullying (29, 73, 81, 86, 101, 122, 124, 126, 127). Some indicate other girls and women as main enforcers of these norms and the most frequent perpetrators of peer victimization (29, 61, 84, 100, 124, 128). Seeing one’s own transgressions of social expectations as the cause of violence may result in greater helplessness and self-blame in relationships and therefore affect well-being and future social interactions (29, 66, 73, 86, 121).

### 5.2 Sexual violence

Quantitative research on sexual victimization face substantive and methodological challenges, such as definitions of sexual violence, measurement tools, and reaching participants. Majority of studies demonstrate that female sex is linked with increased vulnerability to sexual victimization and being autistic female further enhances the risk (113, 114, 129, 130). When no significant differences between females and males on the autism spectrum are observed, it is because the rate of sexual violence reported by autistic males is strikingly high (115, 131). Females on the autism spectrum encounter sexual abuse in adulthood but also below the age of consent, and the frequency of experiencing sexual violence reaches 90% (68). The perpetrators are reported to be both strange men or/and those they were familiar with (29, 68, 80, 122, 132). Autistic females face sexual harassment also in the online world (133). Homosexual orientation may be an additional risk factor for autistic females’ adverse sexual experiences (134). In their narratives, females on the autism spectrum identify their low social skills, gullibility, lack of assertiveness, inadequate body language, and a strong desire to be accepted as factors leading them to be vulnerable to sexual victimization (29, 73, 80, 106, 122, 132). The role of sexual knowledge is ambiguous — low levels of sexual knowledge may increase the risk of engaging in unwanted sexual interactions (131), while having sexual knowledge and the desire to engage in sexual relationships may expose autistic females to abuse, such as exploitation by a sexual partner (129). Being a victim of a sexual assault is linked with PTSD and with re-victimization (68).

## 6 Expectation of rejection

Qualitative analyses of social experiences reveal traces of autistic females’ beliefs that others may reject them. The rejection feared by females on the autism spectrum is twofold. First, they report the anxiety of being discriminated or victimized due to autism stigma. Some feel that negative stereotypes towards autism would affect the way other people interact with them (64, 74, 76, 84, 90, 91, 100, 107, 135–137). Others describe the vigilance related to anticipated negative reactions to their behaviors that differ from social norms (83, 121, 123, 125, 128, 136, 138, 139). Second, autistic females fear the invalidation of their autistic identity and experiences because autism is stereotypically viewed as a male condition (77, 78, 87, 102, 106, 137, 140). The fear of being repelled, mistreated or disbelieved is present in relations with friends (84, 91, 121, 125, 128), partners (132), employers and coworkers (74, 76, 100, 138), healthcare professionals (64, 77, 103, 106, 107, 137), and diagnostic and support services providers (77, 78, 87, 102, 140). In a quantitative study on barriers to formal autism diagnosis, concerns about not being believed, listened to, or being accused of “making up” symptoms were the most prominent barriers, regardless of gender. However, females (and gender-diverse individuals) rated these concerns as more severe than males did (141).

## 7 Concealment and disclosure

For autistic females, concealing their autism is a strategy for “social survival”. They do it in order to avoid anticipated stigmatization (29, 74, 83, 90, 91, 94, 142) and to make the establishment of positive social relations more likely (29, 73, 78, 81, 94, 100, 128). The decision to disclose one’s autistic identity may be comparable to coming out (91). Autistic females disclose their diagnosis to close connections in hopes of building more authentic relationships (74, 76, 78, 83, 84, 91, 132). In formal settings, disclosure is motivated by the need for understanding and necessary accommodations, such as those in schools, workplaces, or healthcare environments (74, 83, 90, 100, 102, 103, 107, 137, 142).

When speaking about the actual experiences of disclosure, autistic females point out both positive and negative consequences. Some of them describe growth in personal relations (78, 83, 91, 132). Other report getting adjustments at school, work or in healthcare facilities (83, 90, 100, 103, 137). Disclosing may give freedom of self-expression and help reduce masking (78, 83, 91). Conversely, sharing the truth is often met with disbelief or dismissal (63, 64, 77, 81, 84, 100, 107, 137, 143), and sometimes with rejection and discrimination because of negative stereotypes concerning autism (90, 100, 102, 107, 132). Negative outness experiences may influence future decisions concerning disclosure (78, 90).

## 8 Internalized stigma

Autistic females report internalized negative judgments related to three main areas where they feel they fail to meet societal expectations. First, they describe self-blame and feelings of being “wrong,” “broken,” “weird,” or generally inferior due to behaviors that deviate from neurotypical norms (73–75, 78, 80, 81, 86, 91, 127, 132, 138). Some report that they had believed their autistic traits rendered them incapable of fulfilling social roles, such as those of a partner (132), parent (102), or employee (100, 138). Some present self-blame about their negative social experiences, including victimization (73, 78, 80, 86, 122). Second, they express feelings of failing to meet expectations of stereotypical femininity (76, 122). Third, they describe how their divergence from male-centered stereotypes of autism contributes to feelings of being “fussy,” “impostors,” or undeserving of support and accommodation services (76, 77, 87, 89).

## 9 Identity and connectedness

Autistic identity can be viewed from a personal as well as a social perspective (144–146). Following Meyer’s conceptualization, we consider autistic identity as a personal identification with the diagnostic label of the autism spectrum, which can be more or less personally important, positive, and integrated with other aspects of one’s self-concept (6). The degree to which an individual identifies with a group of people on the autism spectrum we refer to as community connectedness. It includes a sense of belonging and shared experiences, as well as behavioral involvement in activities to benefit the community (53, 57, 147). As shown below, these phenomena are intertwined.

### 9.1 Autistic identity

Receiving an autism diagnosis enables autistic females to renounce guilt and shame and develop more positive identity (73, 75–77, 79–84, 90, 91, 102, 106, 127, 132, 135, 148). This process often involves rejecting a medical, deficit-centered view of autism and embracing its positive aspects, such as uniqueness, good memory, creativity, empathy, and passion for certain topics (80, 81, 83, 91, 135). An additional aspect may be the opportunity to push back against the adverse impacts of failed gender expectations on personal identity (29, 75, 83, 149). Furthermore, feeling validated in their way of functioning can reduce autistic camouflaging, enabling them to replace a sense of false identity with a more authentic self (73, 128). While females on the autism spectrum underline the importance of autism in their self-image (81, 83), they also point out the way in which it is integrated with other aspects of their personalities, creating complex identities (80, 124, 142).

### 9.2 Community connectedness

Positive peer relationships are vital but challenging for autistic individuals, and loneliness is a common experience (65, 67, 86, 121, 125–128, 139, 150–153). Female peer connections often involve complex social rules, which may create a particularly harmful gap between the social skills of autistic females and the demands of social interactions within their gender group (65, 67, 86, 120, 121, 152, 153). Links between autistic identity and social connectedness are two-way. Positive autistic identity enables one to connect with autistic community (73, 75–77, 102, 106). In turn, connectedness with other autistic individuals enhances appraisal of one’s autistic traits (29, 73–78, 102, 106, 138, 151). It also offers a sense of belonging (29, 73, 75, 77, 78, 80, 81, 84, 102, 135, 136, 142, 143), less effortful social interactions (75, 81, 135, 143), and informational and emotional support (81, 84, 102, 136, 137, 140, 143). Having an authentic and relevant self-image also facilitates connections with neurotypical individuals, improving social relationships and increasing access to social support (77, 86, 106, 135, 138).

The works included in the review from which it was possible to draw information on identity and community connectedness are qualitative studies and do not include cross-gender comparisons. A review of quantitative studies on autistic identity by Davies et al. (145) indicated sparse and inconclusive results on the relationship between autistic identity and sex/gender. Thus, based on the data collected in our review, we can only emphasize that the diagnostic delay in females on the spectrum hinders the development of a positive identity and community connectedness. Some papers included in our review involved samples composed of both females with a formal autism diagnosis and self-diagnosed. Among the studies discussed in this section, only three included female participants without a formal diagnosis. Two of these studies analyzed material posted on blogs and forums, in which verification of diagnosis status would have been impossible anyway (77, 84). The study of Harmens et al. (76) combined self-identified and pending diagnosis participants into one group. The remaining studies, conducted using interviews, required a formal diagnosis. As a result, it is impossible to know whether there are any differences in the experience of autistic identity and community connectivity depending on diagnostic status. At the same time, this indicates the importance of formal diagnosis as validation of females’ autistic selves.

## 10 Discussion

This review aimed to synthesize the social experiences of females on the autism spectrum using the minority stress model as a conceptual framework. Basing on relatable and methodologically sound studies including samples of autistic females, we can conclude that autistic females are disadvantaged compared to two populations within which they are minorities: neurotypical females and autistic males. This disadvantage is of intersectional nature. The social stress encountered by females on the autism spectrum is not a simple sum of burdens common for autistic people and females in general. Females on the autism spectrum also face unique strains arising from the particular content of the stigma towards autistic females: not feminine enough and not properly autistic. This stigma, combined with sexism and broader autism-related stigma, translates into lived experiences of discrimination and victimization, leading to expectations of rejection, concealment and selective disclosure, and internalized stigma. Moreover, these chronic social stressors hinder access to coping resources, such as necessary adjustments and social support.

Minority stress of females on the autism spectrum overlaps with other strains common for females. Pregnancy, childbirth, and menopause are physically and mentally difficult transitional periods requiring medical assistance. These are often the situations that expose autistic females to minority stressors such as discrimination or expectation of rejection, and dilemmas about disclosure.

In summary, the experiences of autistic females go beyond their individual characteristics and life events, and are rooted in societal notions of normality, autism, and femininity. Therefore, the minority stress theory provides a useful lens through which to examine the impact of these experiences on mental health.

Most of the studies included in this review were based on relatively small samples of women on the autism spectrum, which limits the extent to which their findings can be generalized to the broader autistic female population. An additional challenge lies in the complexities of diagnosing autism in women. Access to formal diagnosis is often restricted, leading many individuals to self-identify as being on the spectrum. This self-identified group—those without a formal diagnosis—is frequently underrepresented in research. Furthermore, girls and women who do receive a diagnosis may exhibit a more “male-typical” pattern of autistic traits, as existing diagnostic tools are largely designed around such presentations. Consequently, the picture that emerges from the reviewed literature is inevitably incomplete, particularly with regard to the diversity of the autistic population and variations in diagnostic status.

The shared experiences of minority stress among women on the autism spectrum, along with the associated mental health challenges, may significantly shape the clinical presentation of autism in this population. As highlighted in the review, autistic women are frequently exposed to similar social and cultural pressures, which influence their functioning and may contribute to the development of a distinct female autism phenotype. Research on minority stress in this group offers valuable insights into the mechanisms underlying the masking of autistic traits. The social pressure to conform to normative expectations often compels these individuals to conceal their neurodivergent characteristics—efforts that can carry a substantial cost to their psychological well-being. This dynamic creates a complex interplay in which attempts to avoid stigma and rejection may, paradoxically, lead to negative mental health outcomes.

### 10.1 Directions for future research

Although we retrieved 104 relevant articles concerning social experiences of autistic females, only six explored the relationships between those experiences and mental health. Therefore, we pinpoint this gap as an important direction of future research. Moreover, cross-sectional designs of these studies do not allow for conclusions on causality. The relations can be bidirectional, for instance, higher levels of anxiety may increase one’s vulnerability to violence (66). Longitudinal studies are needed to capture the directions of the impact. Identifying factors that mitigate negative mental health outcomes would be of particular practical and clinical value. These factors could be, for instance, positive autistic identity and social connectedness (53, 57, 154).

Almost all studies included in this review recruited participants without intellectual disability. Thus, the conclusions drawn in this work can only be generalized to this part of the autistic female population. It is important to design studies enabling to explore social experiences of autistic females with intellectual disabilities and the impact of these experiences on mental health and well-being in this group. Using adequate prompts and accessible research tools is a good practice allowing participants with intellectual disability to share their experiences (122).

Sampling presents another challenge in research on females on the autism spectrum. Some studies recruit only formally diagnosed participants, potentially overlooking the experiences of those undiagnosed, a significant portion of the autistic female population (1). To overcome this limitation, other studies recruit participants who identify as autistic, regardless of formal diagnosis. This approach, however, carries the risk of inaccuracies in self-diagnosis. As in both cases researchers rely mostly on self-declaration of one’s diagnostic or identity status, it is important to use psychometric measures to confirm the higher rate of autistic traits. Including the variable of diagnostic status in analyses allows for the identification of potential differences in the experiences of diagnosed and undiagnosed participants.

Most studies included in this review were conducted in Western countries, mainly the USA, UK, and Western Europe. As societal perceptions of the norm (also gender norms) and stigmatizing attitudes are deeply rooted in culture (24, 32), studies on social stress of autistic females conducted in other cultures would allow to identify differences and similarities in this regard (138).

Minority stress affects the autistic population as a whole, regardless of sex or gender (21). Although findings on sex/gender differences in mental health indicate higher prevalence of internalizing symptoms in autistic females than males, they are not unanimous. In addition, the issue of underdiagnosis of depression in males due to its atypical presentation and predominance of externalizing symptoms is becoming more widely recognized (155, 156). As such, the hypothesis that autistic females are more disadvantaged than autistic males warrants testing using tools that examine minority stress variables and account for both internalizing and externalizing disorders.

Lastly, the above-average prevalence of transgender identity among autistic individuals (157–159) highlights the need to differentiate experiences. Reviewed studies adopted different sex/gender terminology and defined the samples studied in varying ways. For this reason, we have standardized the terminology used, without referring to the aspect of gender identity. It should be noted that gender diversity is more often reported by females on the autism spectrum (157–159). Moreover, some of the experiences of autistic females and autistic gender-diverse people may overlap (122, 141). However, the latter group faces unique minority stress (160), of which the manifestations and effects should be explored separately.

### 10.2 Practical implications

Knowledge of risk and protective factors for autistic females’ mental health and well-being is of great importance in clinical practice. It can be used to fine-tune diagnostic and support services, as well as educational and workplace environments in order to counterbalance the challenges met by girls and women on the autism spectrum.

We propose the following measures to improve the situation of females on the autism spectrum:

1. Including information on female presentation of autistic characteristics and camouflaging autism in the curricula of psychological, medical, and pedagogical studies in order to counteract the bias toward autism as a male condition (161–163).
2. Creating accessible and reliable information resources for parents on the characteristics of autism in girls, also taking into account the issues of difficulties in adjusting to gender expectations (164, 165).
3. Using adequate diagnostic tools and in-depth information from diverse sources in the assessment of female individuals, including methods that evaluate camouflaging (166, 167).
4. Establishing post-diagnostic strength-based support programs aimed at increasing knowledge, self-awareness, and positive identity, as well as assisting in the decisions about disclosure. Such interventions can take the form of peer support groups, which can increase social connectedness (168, 169).

Many of autistic females’ experiences of discrimination were encountered in contact with professionals in clinical and educational settings. As the diagnosis of autism spectrum disorder is a key code for autistic females to access resources to cope with detrimental effects of social stressors, the elimination of barriers to receiving it early is a critical issue. Changing the way society perceives females on the autism spectrum is a long-term task; however, raising awareness among researchers and practitioners about the specificity of the female autism experience is within reach.

## Data Availability

The original contributions presented in the study are included in the article/**Supplementary Material**. Further inquiries can be directed to the corresponding author.
