# Correction: Experiences of females on the autism spectrum through the perspective of minority stress theory: a review

**DOI:** 10.3389/fpsyt.2025.1672758

**Published:** 2025-08-14

**Authors:** Aleksandra Grzeszak, Ewa Pisula

**Affiliations:** Department of Health and Rehabilitation Psychology, Faculty of Psychology, University of Warsaw, Warsaw, Poland

**Keywords:** autism, females, life experiences, mental health, minority stress, social experiences, stigma

In the published article, there was an error in the **Funding** statement. The statement omitted funds obtained during the publication process for the Article Processing Charge. The correct **Funding** statement appears below:

“The author(s) declare that financial support was received for the research and/or publication of this article. This work was supported by the Faculty of Psychology, University of Warsaw, from the funds awarded by the Ministry of Science and Higher Education in the form of a subsidy for the maintenance and development of research potential in 2024 (501-D125-01-1250000, zlec. 5011000193). The funds covered the cost of language proofreading of the article. The Article Processing Fee was subsidized in the internal grant system within the programme of the Ministry of Science and Higher Education “Initiative of Excellence - Research University” at the University of Warsaw, Actions I.2.4 (PSP 501-D125-20-0001241) and IV.4.1 (PSP 501-D125-20-0004410).”

The original version of this article has been updated.

