# Supplementary material for: Experiences of females on the autism spectrum through the perspective of minority stress theory: a review
Source: Front Psychiatry. 2025 Jul 18;16:1578963. doi: 10.3389/fpsyt.2025.1578963 (PMC12314820; doi:10.3389/fpsyt.2025.1578963)
Supplement: Supplementary file 1 [file DataSheet1.pdf]

Supplementary Material – Search Strings

Database	Search String	Additional Filters (if not applied in query)
Web of Science	(((TS=(("autistic women" OR "women on the autism spectrum" OR "women with autism" OR "women with ASD" OR "women with HFA" OR "women with Asperger's" OR "neurodivergent women" OR "autistic females" OR "females on the autism spectrum" OR "females with autism" OR "females with ASD" OR "females with HFA" OR "females with Asperger's" OR "neurodivergent females" OR "autistic girls" OR "girls on the autism spectrum" OR "girls with autism" OR "girls with ASD" OR "girls with HFA" OR "girls with Asperger's" OR "neurodivergent girls") OR (("gender difference*" OR "sex difference*") AND (autis* OR Asperger* OR neurodivergent)))) AND TS=(experience* OR stigma* OR label* OR stereotype* OR prejudice* OR rejection OR discrimination OR inequit* OR inequalit* OR sexis* OR victimization OR violence OR bullying OR concealment OR disclosure OR identity))) NOT TS=(review OR commentary OR editorial OR opinion)	Publication years: 2013-2024<br>Document types: article<br>Languages: English
Scopus	TITLE-ABS-KEY (("autistic women" OR "women on the autism spectrum" OR "women with autism" OR "women with ASD" OR "women with HFA" OR "women with Asperger’s" OR "neurodivergent women" OR "autistic females" OR "females on the autism spectrum" OR "females with autism" OR "females with ASD" OR "females with HFA" OR "females with Asperger’s" OR "neurodivergent females" OR "autistic girls" OR "girls on the autism spectrum" OR "girls with autism" OR "girls with ASD" OR "girls with HFA" OR "girls with Asperger’s" OR "neurodivergent girls") OR (("gender difference*" OR "sex difference*") AND (autis* OR asperger* OR neurodivergent))) AND TITLE-ABS-KEY (experience* OR stigma* OR label* OR stereotype* OR prejudice* OR rejection OR discrimination OR inequit* OR inequalit* OR sexis* OR victimization OR violence OR bullying OR concealment OR disclosure OR identity) AND NOT TITLE-ABS-KEY (review OR commentary OR editorial OR opinion) AND PUBYEAR > 2012 AND PUBYEAR < 2025 AND (LIMIT-TO (SRCTYPE , "j")) AND (LIMIT-TO (DOCTYPE , "ar")) AND (LIMIT-TO (LANGUAGE , "English"))	
